# “Stay at Home” in Italy during the COVID-19 Outbreak: A Longitudinal Study on Individual Well-Being among Different Age Groups

**DOI:** 10.3390/brainsci11080993

**Published:** 2021-07-27

**Authors:** Alessandro Quaglieri, Giulia Lausi, Angelo Fraschetti, Jessica Burrai, Benedetta Barchielli, Alessandra Pizzo, Pierluigi Cordellieri, Luigi De Gennaro, Maurizio Gorgoni, Fabio Ferlazzo, Stefano Sdoia, Pierpaolo Zivi, Anna Maria Giannini, Emanuela Mari

**Affiliations:** 1 Department of Psychology, “Sapienza” University of Rome, Via dei Marsi 78, 00185 Rome, Italy; giulia.lausi@uniroma1.it (G.L.); angelo.fraschetti@uniroma1.it (A.F.); jessica.burrai@uniroma1.it (J.B.); alessandra.pizzo@uniroma1.it (A.P.); pierluigi.cordellieri@uniroma1.it (P.C.); luigi.degennaro@uniroma1.it (L.D.G.); maurizio.gorgoni@uniroma1.it (M.G.); fabio.ferlazzo@uniroma1.it (F.F.); stefano.sdoia@uniroma1.it (S.S.); pierpaolo.zivi@uniroma1.it (P.Z.); annamaria.giannini@uniroma1.it (A.M.G.); e.mari@uniroma1.it (E.M.); 2 Department of Dynamic and Clinical Psychology and Health Studies, “Sapienza” University of Rome, Via degli Apuli 1, 00185 Rome, Italy; benedetta.barchielli@uniroma1.it

**Keywords:** coronavirus, perceived stress, emotional state, young adults, social distance

## Abstract

The restrictions imposed by the Italian government because of the coronavirus outbreak have been shown to be demanding on the Italian population. Data were collected at four different time points from 29 March 2020 to 3 May 2020 and during the final follow-up survey on 12 October 2020. In the present study, we provided longitudinal evidence on the relationship between the lockdown and mental health dimensions, such as emotional state, perceived stress, and time perspective, for three age groups. The results allowed us to observe their psychological status from different perspectives at five different time points. Notably, a negative effect of the lockdown individual well-beings emerged as a trend, and differences in individual adaptation strategies to a prolonged stressful situation were observed at the follow-up. Indeed, pairwise comparisons between age groups showed that the young adult group (18–23 years old) seemed to be the most psychologically affected by the lockdown. The findings are discussed according to the most recent literature on the topic. To the best of our knowledge, this is one of the first longitudinal studies carried out in Italy concerning the general psychological effects of the coronavirus lockdown.

## 1. Introduction

At the beginning of 2020, a pandemic caused by the SARS-CoV-2 infection [1] spread in Italy and created an unprecedented health situation. In the 48 h before the decree that established the lockdown in Italy (10 March 2020), there was an increase in the number of total deaths of nearly 100% (i.e., total number of deaths registered by the healthcare system) [2]. This increase, according to Perone [3], was mainly caused by massive stress on the Italian health system.

The lockdown imposed by the Italian government involved severe restrictions on people’s movement, which was suddenly allowed only for reasons related to health or essential activities and services (e.g., going to hospitals, pharmacies, or supermarkets). While the lockdown measures were intended to flatten the contagion curve, positive or negative side effects on the social, economic, and psychological aspects of everyday life must also be considered. In fact, some people may benefit from the suspension of activities and from increased time with family, exhibiting reduced stress levels and improved mental health and well-being; on the other hand, for others, anxiety and stress could increase because of infection risk [4]. A number of studies have reported a positive relationship between an increase in contagions and the levels of anxiety, depressive and posttraumatic stress disorder symptoms, psychological distress, and stress, both in the general population [5,6,7,8,9,10,11,12] and in clinical populations [13]. For instance, a recent study [14] of 439 people showed an increase in stress and depression over the 2-month lockdown in Italy. It was also found that a negative affect and detachment were associated with higher levels of both depression and stress, especially for those whose levels that were higher at the beginning of the lockdown period. Additionally, it has been argued that the length of lockdown can have both medium- and long-term economic and social effects, which are strictly associated with frustration, boredom, and inadequate information [6]. Another psychological dimension related to individual well-being is the time perspective [15]. It has been described as a personal time orientation linked to the past, present, or future [16]. Different studies have investigated the future positive perspective in relation to COVID-19-preventing behaviors [17,18]. Additionally, a study has highlighted the importance of investigating the future time perspective because of its moderating role between risk perception and depression [19].

All of these issues have also been considered important stressors affecting the well-being of the population. Furthermore, studies have also reported individual differences in the psychological effects of lockdown. For example, Bruine de Bruin [20] found that older adults showed greater emotional well-being when facing the threat of COVID-19 than younger adults. Roma et al. [14] also reported a negative correlation between age and stress levels during the lockdown, with emerging adults reporting higher stress levels than older adults.

While longitudinal studies are the preferable design to investigate the effects of a long-lasting condition such as lockdown, during the present pandemic, they have been relatively scarce for obvious reasons. Notable exceptions exist, however. For instance, several studies [12,21,22,23,24] have considered at least three data points. To the best of our knowledge, very few longitudinal studies have investigated the consequences of the lockdown in Italy within the restriction period [25]. Notably, all the published studies on the psychological effects of the pandemic obviously lack a baseline measure, which complicates the interpretation of the results. Furthermore, they also lack a follow-up post-lockdown measure that could be used to both interpret the data collected during the lockdown and estimate the duration of the observed effects.

Therefore, in the present study, we surveyed the mental health status of Italians at four time points during the lockdown in Italy (from the end of March to the end of May 2020) and at the end of October 2020 (follow-up). Specifically, adopting Selye’s [26] theory of stress, we hypothesized that, during the lockdown, individuals experienced the different phases of “general adaptation syndrome” (GAS). First, the stressful event (i.e., the COVID-19 pandemic) caught them off-guard and induced an alarm reaction (i.e., the alarm phase) in individuals who attempted to maintain homeostasis by resisting (i.e., the resistance phase); finally, exhaustion occurred if the organism was unable to return to a pre-alarm level of homeostasis (i.e., the exhaustion phase). According to this theory, this longitudinal study aimed to explore the impact of COVID-19 as a stressful event, consistent with Selye’s model, hypothesizing that the trend in five time-point scores could reflect the stages of the GAS model, i.e., the alarm reaction to the pandemic event during the first data collection followed by stasis (i.e., resistance) during the next three lockdown observations and a return to homeostasis or exhaustion during the follow-up.

We investigated the psychological effects of the COVID-19 outbreak by assessing individuals’ emotional states (i.e., positive and negative emotional affects), perceived stress, and future perspectives (i.e., attitudes and concerns about the future) during the confinement imposed by the Italian government. In such an uncertain situation, people may be expected to experience high levels of anxiety, as well as reduced positivity and confidence about the future; however, adaptive coping strategies and a positive orientation toward the future help reduce those negative effects, while a lack leads to prolonged stress and an exhaustion phase [26,27,28], which may result in the chronicity of depression and anxiety symptoms and decreased stress tolerance [29].

Although the pandemic may affect both young and older adults, age may play a pivotal role in determining the degree of impact on psychological well-being. Indeed, social distancing, the limitation of social activities, and stay-at-home policies have all led to a strong reduction of nonvirtual connections with peers and friends, which, in turn, may lead to increased psychiatric symptoms, especially in young people [30,31].

Hence, the current longitudinal study investigated the impact of the Italian lockdown on the individual well-being of people of different ages at five different time points, including a follow-up during the COVID-19 pandemic. The term individual well-being embraces a wide range of connotations that differ according to the context and both professional and personal perspectives [32]. The type of individual well-being investigated in this study considers both subjective and psychological dimensions. In fact, psychological well-being refers to different levels of positive functioning (e.g., relationships with others and self-referent attitudes), while subjective well-being reflects subjective evaluations of different aspects of one’s quality of life (e.g., psychological and physical health) [32].

From these premises, we hypothesized the following:

**Hypothesis 1 (H1).**  *Within the whole sample, the GAS model pattern could be observed (e.g., an early increase in, followed by stabilization of, the anxiety and stress levels).*

**Hypothesis 2 (H2).**  *Younger adults may have been affected more by the lockdown measures, exhibiting higher anxiety, stress, and negative affect scores but lower future perspective, positive attitude, and coping strategy scores than older adults.*

## 2. Materials and Methods

### 2.1. Participants

A total of 123 participants were involved in the study. The inclusion criteria were: having reached the age of majority (18 years) and having electronic devices and an internet connection available to complete the questionnaire. Participants who did not participate in all stages of the research were excluded. Medical histories were not investigated.

### 2.2. Procedure and Materials

**Figure 1:**
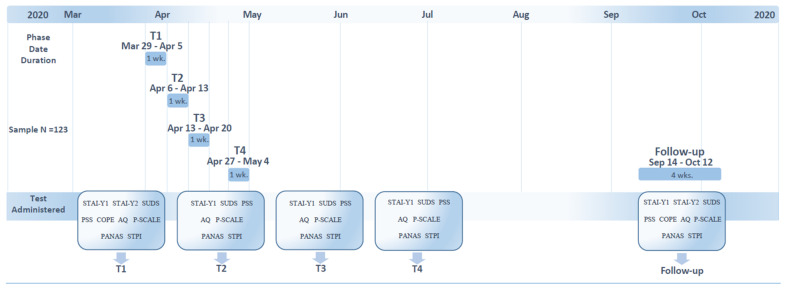
Summary of the survey dates and tests administered at each time point.

We collected repeated measures related to individual well-being through an online survey via the Qualtrics platform. This longitudinal study (Figure 1) was launched on 29 March 2020 (very close to the beginning of the isolation measures in Italy) and concluded on 12 October 2020. We deployed our first survey on 29 March 2020, and the survey remained open until 5 April 2020. This first survey (Time 1; T1) was distribution by social networks and the university’s official website. During this phase, participants agreed to participate by signing a digital informed consent form and provided their email address stating their intention to answer future surveys. Participants were informed that the data collected were anonymous and were not shared outside the research group. Seven to fourteen days later, from 6 April 2020 to 12 April 2020, depending on the “response day” during T1, the same participants responded to a second survey (Time 2; T2). A third survey was launched on 13–19 April 2020 (Time 3; T3), and 15–20 days later, a fourth survey was launched from 27 April to 3 May 2020 (Time 4; T4). On 4 May, the lockdown was lifted, and the restrictions in Italy started to be gradually lightened. A final follow-up (FU) survey was administered between 14 September and 12 October 2020. This study was conducted in accordance with the ethical standards of the Helsinki Declaration and was approved by the Institutional Review Board of the Department of Psychology of Sapienza University of Rome (prot. N 577 of 28/03/2020).

#### 2.2.1. State-Trait Anxiety Inventory (STAI-Y1; STAI-Y2)

The STAI-Y is a 40-item four-point self-reported questionnaire using a Likert scale ranging from 1 to 4 that was designed to assess and differentiate anxiety as a state (i.e., a feeling of insecurity and helplessness; STAI-Y1) and as a trait (i.e., a tendency to perceive stressful situations as dangerous and threatening; STAI-Y2) [33,34]. The STAI-Y1 scale was administered at each session, while the STAI-Y2 was administered at T1 and the follow-up session. Higher scores indicate a higher level of both dimensions of anxiety. The STAI-Y1 showed internal consistency, with a Cronbach’s alpha of 0.84 and an alpha of 0.86.

#### 2.2.2. Subjective Units of Distress Scale (SUDS)

The SUDS is a self-reported questionnaire ranging from 0 to 10 that measures the intensity of a negative emotion and disturbance experienced by an individual. A value of 0 represents no discomfort (e.g., peace and total relief), and a value of 10 represents the most intense disturbance [35]. This indicator was used in each session. For this survey, the Cronbach’s alpha was 0.88.

#### 2.2.3. Perceived Stress Scale (PSS)

The PSS-10 is a five-point scale (0–4) measuring the perception of stress [36,37]. It allows an investigation of how unpredictable, uncontrollable, and overloaded participants experience their own lives. This scale was administered at each session. Scores ranging from 0 to 13 indicated low stress, those from 14 to 26 indicated moderate stress, and scores from 27 to 40 indicated high perceived stress. For this survey, the Cronbach’s alpha was 0.88.

#### 2.2.4. Brief COPE (COPE) Inventory

The Brief COPE is a 28-item questionnaire with a four-point scale (1–4). This scale allows the assessment of different coping strategies or regulating cognitions in response to stressful events [38,39]. The scale evaluates individuals’ primary coping styles and is composed of two main factors (i.e., avoidant and approach coping) and two independent subscales (i.e., humor and religion). It was administered during the first and follow-up sessions. Higher scores indicate an increased utilization of that specific coping strategy. The Cronbach’s alpha for the total scale was adequate, because all values exceeded the minimum value of 0.60 (i.e., avoidant: 0.78, approach: 0.67, humor: 0.64, and religion: 0.85).

#### 2.2.5. Aggression Questionnaire (AQ)

The AQ is a 23-item five-point scale (1–5) measuring an individual’s aggressive responses and the ability to manage them [40,41]. This scale consists of a three-factor structure: hostility (AQ-HO), physical aggression (AQ-PA), and verbal aggression (AQ-VA). This scale was administered at each session. Higher scores indicate higher aggressive behavior. The internal consistency coefficients were as follows: anger (0.68), physical aggression (0.73), verbal aggression (0.61), and hostility (0.74).

#### 2.2.6. Positivity Scale (P-Scale)

The P-Scale is an eight-item questionnaire with a five-point scale (1–5), and it is a widely used instrument to assess individuals’ tendency to approach and experience their lives with a positive attitude [42]. The scale was administered at each session. Higher scores indicate a higher positive attitude. The Cronbach’s alpha was 0.93.

#### 2.2.7. Positive and Negative Affect Schedule-Trait (PANAS)

The PANAS consists of two 10-item questionnaires with five-point scales ranging from 1 to 5. It is a widely and frequently used scale developed to investigate the positive affects (PA) and negative affects (NA) [43,44]. This scale was administered at each session. Higher scores indicate a greater experience of specific affects for each dimension. The Cronbach’s alpha value for the PA dimension was 0.86, and the alpha for the NA dimension was 0.88.

#### 2.2.8. Stanford Time Perspective Inventory (STPI)—Short Form

The STPI is a 22-item self-reported questionnaire with a five-point scale ranging from 1 to 5. Originally developed by Zimbardo and Boyd [45], it investigates personal time orientation, including three factors: present–hedonistic, present–fatalistic, and future time perspectives. In this study, the Italian short-form version by D’Alessio et al. [46] was administered at each session. A higher score indicated a greater individual temporal perspective. The future dimension showed a Cronbach’s alpha of 0.67, the present–hedonistic dimension had an alpha of 0.54, and the alpha obtained for the present–fatalistic dimension was the lowest at 0.48.

### 2.3. Data Analysis

Statistical analyses were performed using the Statistical Package for the Social Sciences (SPSS; version 25.0; IBM SPSS, Armonk, NY, USA). Descriptive statistics were reported for gender, age, education, marital status, employment, house size (square meters), number of cohabitants, pet ownership, and availability of outdoor spaces (Table 1). The questionnaire scores were analyzed in different 3 × 5 mixed ANOVA designs, with age as a between-subjects factor (3 groups: 18–23, 24–36, and +37 years old) and session as a within-subjects factor (5 levels: T1, T2, T3, T4, and FU). The STAI-Y2 and COPE questionnaires were analyzed in 3 × 2 mixed ANOVAs, with age as a between-subjects factor and session as a within-subjects factor (2 levels: T1 and FU). We conducted a post hoc analysis with G*Power software [47]; assuming a conventional effect size of 0.31 [48] and considering 123 individuals divided into three homogeneous groups across five repeated measurements, the results showed a power of 0.88 (two-sided alpha = 0.05).

The partial eta squared (i.e., ηp^2^) was used to determine the effect size. A value of 0.01 ηp^2^ indicates a small effect, a ηp^2^ of 0.06 indicates a medium effect, and a ηp^2^ of 0.14 indicates a large effect size. The Greenhouse–Geisser correction was applied in cases of significant violations of the sphericity assumption, and post hoc analyses using the Bonferroni correction for alpha inflation due to multiple testing were performed (implemented in SPSS). No missing data were found, as each item required a response to continue with the next step of the questionnaire. The normality of all the data was verified, and all statistical analyses were performed on deidentified data.

## 3. Results

### 3.1. Participants

A total of 123 participants were involved in the study; 26% of the participants were male, and the ages of the participants ranged from 18 to 74 (M = 33.9, SD = 15.52). According to the aims of our study, three age groups were investigated: emerging adults, young adults, and middle adults. The sample included 46 emerging adult participants (18–23 years old; M = 21.67, SD = 1.19), 38 young adults (24–36 years old; M = 27.0, SD = 2.92), and 39 middle adults (37+ years old; M = 55.15, SD = 8.44). A total of 95.9% of the respondents were affected by the isolation measures (e.g., students, employees, freelancers, and workers), and 4.1% were exempted (e.g., police forces and healthcare professions). Ninety-five percent of the participants lived with other people, and 48% had a domestic animal/s. Sixty-five percent of the sample lived in an 80+ sqm house, and 95.9% had an availability of outdoor spaces. The full sociodemographic characteristics are described in Table 1.

### 3.2. Emotional State

**Figure 2:**
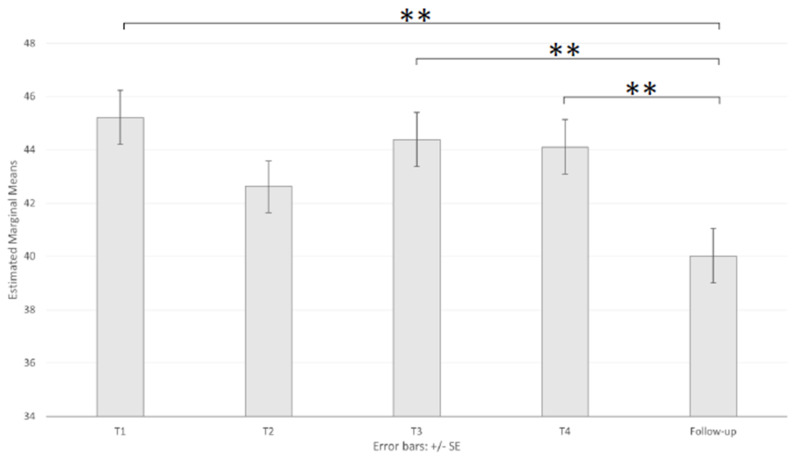
Main effect of session on the State Anxiety Inventory. Note: ** *p* < 0.01.

The ANOVA of the STAI-Y1 mean scores revealed a significant main effect of the sessions (F _(3.245, 389.359)_ = 8.28, *p* < 0.001, ηp^2^ = 0.065), showing significantly lower mean scores on the FU (40.10) than the other sessions, with the notable exception of T2 (45.54, 42.85, 44.56, and 44.26 for T1, T2, T3, and T4, respectively; Table 2 and Figure 2). The ANOVA also showed a significant main effect of age (F _(2, 120)_ = 4.73, *p* < 0.05, ηp^2^ = 0.073), which was explained by a significantly higher mean score in the 18–23-year-old group (M = 46.54, SD = 1.29) than in the 37+-year-old group (M = 41.04, SD = 1.40, *p* < 0.05). The interactions did not reach statistical significance (F _(6.489, 389.359)_ = 1.20, *p* = 0.304).

In contrast, the ANOVA of the STAI-Y2 mean scores did not reveal a significant main effect of the session (F _(1, 120)_ = 1.46, *p* = 0.229). Instead, there was the significant main effect of age (F _(2, 120)_ = 11.14, *p* < 0.001, ηp^2^ = 0.157), with the 18–23-year-old group exhibiting significantly higher mean scores (M = 49.34, SD = 1.34) than both the 24–36-year-old (M = 43.42, SD = 1.47) and 37+-year-old groups (M = 40.22, SD = 1.45, *p* < 0.05 and *p* < 0.001, respectively; Table 2); however, the interaction was not significant (F _(2, 120)_ = 1.01, *p* = 0.366). The difference in trait anxiety among the age groups may explain the main effect of age on the state anxiety.

**Figure 3:**
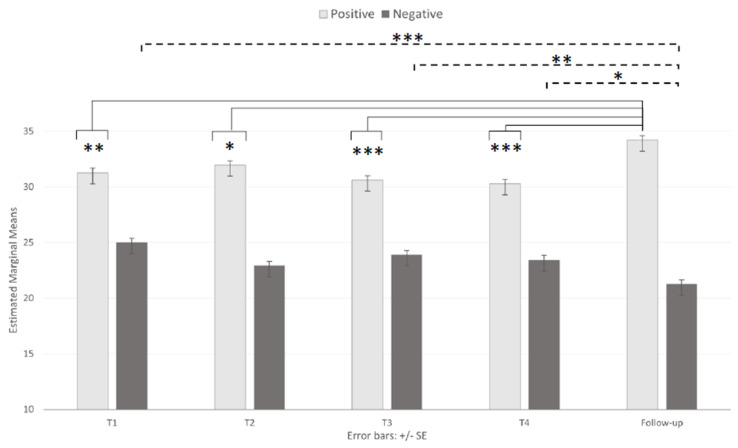
Main effect of session on the positive and negative affects schedule. Note: * *p* < 0.05, ** *p* < 0.01, and *** *p* < 0.001.

The mean scores for both the positive affect (PA) and negative affect (NA) dimensions in the PANAS were analyzed by two separate ANOVAs. Both analyses revealed significant main effects of the session (F _(2.802, 336,244)_ = 11.49, *p* < 0.001, ηp^2^ = 0.087 and F _(3.398, 407,728)_ = 9.48, *p* < 0.001, ηp^2^ = 0.073 for the positive and negative affect dimensions, respectively); nonsignificant main effects of age (PA = 30.33, 31.97, and 32.72, respectively, for 18–23, 24–36, and 37+; NA = 24.62, 23.17, and 22.12, respectively, for 18–23, 24–36, and 37+; *p* > 0.05); and nonsignificant interactions (F _(5.604, 336,244)_ = 1.67, *p* = 0.131 and F _(6.795, 407,728)_ = 0.41, *p* = 0.89) for the positive and negative affect dimensions, respectively. Multiple comparisons of the main effects of the session (Table 2 and Figure 3) showed that the participants exhibited significantly higher (*p* < 0.05) mean scores at the FU than in all other sessions (31.27, 31.96, 30.63, and 30.28 for T1, T2, T3, and T4, respectively) on the PA scale and significantly lower (*p* < 0.05) mean scores at the FU than at T1, T3, and T4 (25.01, 23.90, and 23.45, respectively) on the NA scale.

**Figure 4:**
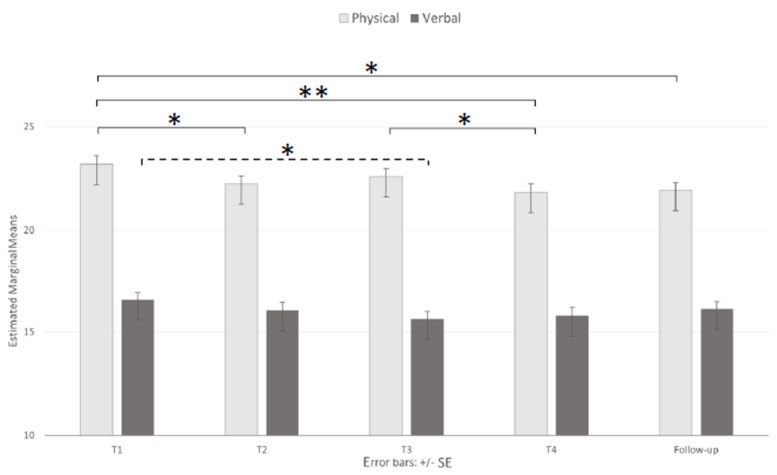
Main effect of session on physical and verbal aggression. Note: * *p* < 0.05 and ** *p* < 0.01.

Three separate ANOVAs were conductedr on the mean scores of each dimension of the AQ questionnaire. An analysis of the AQ-PA dimensions (Figure 4) revealed significant main effects of both session (F _(3.538, 424.592)_ = 5.74, *p* < 0.001, ηp^2^ = 0.046) and age (F _(2, 120)_ = 10.56, *p* < 0.001, ηp^2^ = 0.150) but no significant interaction (F _(7.077, 424.592)_ = 1.25, *p* = 0.267). Specifically, the mean scores (23.35, 22.39, 22.71, 21.95, and 22.09 for T1, T2, T3, T4, and FU, respectively) were significantly different between T1 and T2 (*p* < 0.05), T1 and T4 (*p* < 0.01), T1 and the FU (*p* < 0.05), and T3 and T4 (*p* < 0.05). Post hoc tests for the effects of age revealed higher mean scores in the 18–23-year-old group (M = 24.45, SD = 0.60) than both the 24–36-year-old (M = 22.25, SD = 0.66, *p* < 0.05) and 37+-year-old (M = 20.43, SD = 0.65, *p* < 0.001) groups.

Similarly, an analysis of the AQ-VA dimensions (Figure 4) showed a significant main effect of the session (F _(3.447, 413.671)_ = 3.13, *p* < 0.01, ηp^2^ = 0.025), as the participants showed significantly higher (*p* < 0.05) mean scores at T1 than at T3 (16.62 and 15.69, respectively) and a significant main effect of age (F _(2, 120)_ = 3.30, *p* < 0.05, ηp^2^ = 0.052), with the 18–23-year-old group (M = 17.11, SD = 0.67, *p* < 0.05) exhibiting significantly higher mean scores than the 37+-year-old group (M = 14.61, SD = 0.73, *p* < 0.05). Even in this case, the interactions were nonsignificant (F _(6.895, 413.671)_ = 1.63, *p* = 0.126).

Finally, an ANOVA of the AQ-HO mean scores showed no significant main effect of session (F _(3.138, 376.586)_ = 0.649, *p* = 0.591) and no significant interactions (F _(6.276, 376.586)_ = 1.09, *p* = 0.366). However, there was a significant main effect of age (F _(2, 120)_ = 6.37, *p* < 0.01, ηp^2^ = 0.096). The post hoc tests showed that the mean scores in the 18–23-year-old group (M = 21.20, SD = 0.76) were significantly higher than those in the 37+-year-old group (M = 17.17, SD = 0.83, *p* < 0.01).

With respect to the emotional state dimensions, our results showed different patterns across the five time points. The STAI scores showed partial recovery at the FU, and the PA dimension score was reduced in the middle of the lockdown (at T3 and T4) and increased at the FU; however, the NA dimension score showed a progressive decrease from T3 to the FU. Finally, the AQ dimensions showed differences between the middle time points and the FU and between the middle time points and T1 for both the AQ-PA and AQ-VA dimensions. Overall, regarding the differences among group ages, the emerging adults reported higher scores than the other groups across all emotional state dimensions.

Similarly, an analysis of the AQ-VA dimensions (Figure 4) showed a significant main effect of session (F _(3.447, 413.671)_ = 3.13, *p* < 0.01, ηp^2^ = 0.025), as the participants showed significantly higher (*p* < 0.05) mean scores at T1 than at T3 (16.62 and 15.69, respectively), and a significant main effect of age (F _(2, 120)_ = 3.30, *p* < 0.05, ηp^2^ = 0.052), with the 18–23-year-old group (M = 17.11, SD = 0.67, *p* < 0.05) exhibiting significantly higher mean scores than the 37+-year-old group (M = 14.61, SD = 0.73, *p* < 0.05). Even in this case, the interactions were nonsignificant (F _(6.895, 413.671)_ = 1.63, *p* = 0.126).

### 3.3. Perceived Stress

**Figure 5:**
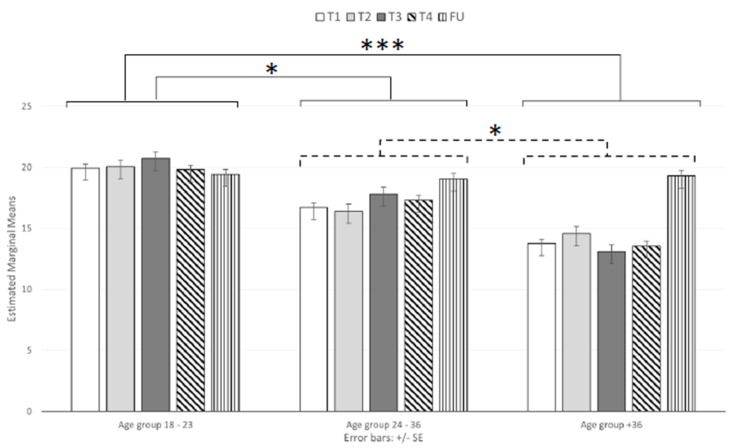
Interaction effect of the age group on the perceived stress scale. Note: * *p* < 0.05 and *** *p* < 0.001.

We analyzed the total mean scores of the PSS-10 and found a significant main effect of session (F _(3.331, 399.780)_ = 9.95, *p* < 0.001; ηp^2^ = 0.077), as the mean scores were higher at the FU than at the other sessions (16.98, 17.20, 17.41, and 17.07 for T1, T2, T3, and T4, respectively; Table 3). The ANOVA also showed a significant main effect of age (F _(2, 120)_ = 15.83, *p* < 0.001; ηp^2^ = 0.209; Table 3 and Figure 5), and the post hoc tests showed that the mean scores were higher in the 18–23-year-old group (M = 19.99, SD = 0.62) than in both the 24–36-year-old (M = 17.46, SD = 0.68, *p* < 0.05) and 37+-year-old groups (M = 14.87, SD = 0.67, *p* < 0.001) and higher in the 24–36-year-old group than in the 37+-year-old group (*p* < 0.05).

The results also highlighted a significant interaction effect (F _(6.663, 399.780)_ = 7.38, *p* < 0.001; ηp^2^ = 0.110). Specifically, the post hoc tests showed that mean scores at T1, T2, and T4 were significantly higher for both the 18–23-year-old and 24–36-year-old age groups than for the 37+-year-old group.

In addition, the scores of the 18–23-year-old group also differed from those of the 24–36-year-old group at T1 and T2 (*p* < 0.05 and *p* < 0.001 for T1 and T2, respectively). Interestingly, except for at the FU, the scores of the youngest groups significantly differed from those of the oldest group at all time points. However, among the three groups, the emerging adult group exhibited higher scores at all lockdown time points, including the FU.

**Figure 6:**
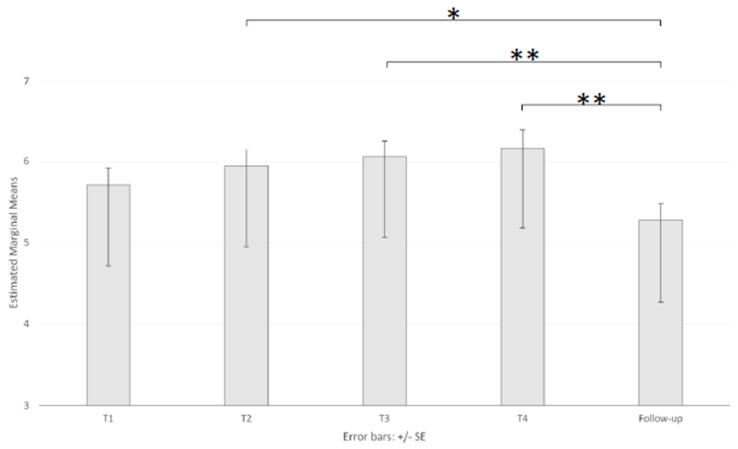
Main effect of session on the Subjective Units of Distress Scale. Note: * *p* < 0.05 and ** *p* < 0.001.

The ANOVA of the SUDS mean scores showed a significant main effect of session (F _(3.074, 368.880)_ = 7.4, *p* < 0.001, ηp^2^ = 0.058). Pairwise comparisons showed that the mean scores (5.75, 5.97, 6.11, 6.22, and 5.28 for T1, T2, T3, T4, and FU, respectively; Table 3 and Figure 6) at the FU were significantly lower than those at T2 (*p* < 0.05), T3 (*p* < 0.01), and T4 (*p* < 0.01). There was no significant main effect of age (F _(2, 120)_ = 2.87, *p* = 0.060) and a nonsignificant interaction (F _(6.148, 368.880)_ = 1.95, *p* = 0.069).

Separate ANOVAs were conducted for the two factors of the COPE. With respect to the avoidant dimension, there was a significant main effect of session (F _(1, 120)_ = 5.57, *p* < 0.05, ηp^2^ = 0.044), a significant main effect of age (F _(2, 120)_ = 6.75, *p* < 0.01, ηp^2^ = 0.101), and a nonsignificant interaction (*p* > 0.05). Specifically, participants exhibited a higher mean score at T1 than at the FU (21.78 and 21.04, respectively; Table 3). The post hoc analysis for age showed that the mean score of the 18–23-year-old group was higher than that of the 37+-year-old group (*p* < 0.01).

In contrast, the analysis of the approach dimension did not reveal any statistically significant effects (*p* > 0.05 for all effects).

Two separate ANOVAs were also conducted for the two independent subscales of the COPE. The analysis of the humor subscale showed no statistically significant effects (*p* > 0.05), whereas the analysis of the religion subscale revealed a significant main effect of age (F _(2, 120)_ = 3.76, *p* < 0.05, ηp^2^ = 0.059), with the post hoc tests showing that the mean score of the 18–23-year group (M = 2.52, SD = 0.19) was lower than the mean score of the 37+-year-old group (M = 3.27, SD = 0.21, *p* < 0.05, Table 3). No other significant effects were found (*p* > 0.05).

The SUDS scores showed a partial recovery in the FU session, while the PSS-10 scores were higher at the FU session than at the other sessions for the 24–36-year-old and 37+-year-old age groups, providing evidence for increased perceived stress. In addition, the PSS-10 data showed an influence of age on the trend observed. Indeed, except for at the FU, when the participants showed similar total PSS-10 scores, emerging adults exhibited higher scores than the other two groups. Regarding the COPE inventory, the young group scored higher on the avoidant dimension than the other groups, while the middle adult sample scored higher on the religion dimension.

### 3.4. Future Perspective

Concerning the positivity scale, we found a significant effect of session (F _(3.261, 391.265)_ = 3.67, *p* < 0.05, ηp^2^ = 0.030), with the mean score being significantly higher at T1 than at T3 and T4 (28.25, 28.05, 27.41, 26.98, and 27.59, for T1, T2, T3, T4, and FU, respectively; Table 4). Both the main effect of age and the interaction were nonsignificant (*p* > 0.05).

Regarding the STPI, separate ANOVAs were conducted for the future, fatalistic, and hedonistic dimensions. The mean scores on the future subscale showed a significant main effect of session (F _(3.569, 428.283)_ = 2.88, *p* < 0.05), with a significant difference between T4 and the FU (32.91, 32.70, 32.92, 32.31, and 33.52 for T1, T2, T3, T4, and FU, respectively; Table 4); no other significant effects were observed (*p* > 0.05). No significant main effects or interactions were found for either the fatalistic or hedonistic dimensions (*p* > 0.05).

In summary, the data obtained from the positivity scale and the STPI showed that participants’ tendency to view life and experiences through a positive lens was reduced during the late phase of the lockdown.

## 4. Discussion

As expected, our results overall showed a time-dependent adaptation of individual well-being to the lockdown conditions, although this adaptation was slightly different for the three investigated dimensions (emotional state, perceived stress, and future perspective). In summary, individuals reported higher levels of anxiety during the first four time points (lockdown) than at the FU, higher physical and verbal aggression during the early phase of the lockdown (first time point) than at the two other time points during the lockdown and the FU, and a progressive reduction in NA scores during the lockdown supported by an increase in PA scores, especially at the FU. Finally, the participants showed a mild decrease in positive feelings and a reliance on the future in the final phase of the confinement.

We first found that participants exhibited higher levels of anxiety throughout most of the lockdown period investigated. This is unsurprising, since fears and worries, especially those related to health and the economy, were prominent during the period in which the present study focused. According to the current literature [49], our study suggests that high levels of anxiety were experienced beginning in the early phase of the lockdown and that the anxiety levels were decreased at the FU, when the individuals adapted to the condition. Anxiety was found to be the most frequently experienced mood during the pandemic [50,51]. In fact, both the emotional stress and the unusual emotional responses related to the COVID-19 outbreak may exacerbate previous psychiatric conditions or precipitate their symptomatologies.

Mood and negative affects are often linked to the emotions of irritation, agitation, and anger in a threatening situation [52]. The results showed that the AQ scores were higher in the early sessions than at the FU for both physical and verbal aggression. The uncertainty and lack of control that individuals experienced in the early stages of the pandemic may have been related to the likelihood of being infected and the potential severity of the infection, as well as the expectation of resuming a “normal life”. This state of uncertainty may have caused stress, leading people to be more likely to exhibit aggressive behavior [53]. In addition, it is possible to hypothesize that the first quarantine period may have been characterized by different types of frustrations. Several major theories of aggression consider frustration to be blocking goal-directed actions and to be a powerful instigator of aggression [54]. A major frustration experienced during the onset of the pandemic was being forced to spend most of the time at home, which, in extreme cases, led to increased rates of aggressive and violent behaviors (e.g., intimate partner violence) [55]. According to different studies, forced cohabitation with abusive partners may have exacerbated individual and social vulnerability, limiting coping skills and reliance [56,57]. The social distancing rules may have also promoted a sense of loneliness and social exclusion that may have threatened another vital motivation, “the need to belong”.

The COVID-19 pandemic, which caused an extreme disruption of normal life and uncertainty for the future, may have generated feelings of frustration/aggression, in line with Berkowitz’s revised model [54]. It seems reasonable to assume that younger people suffered more from the drastic effects of the pandemic on their lives with regard to employment, education, mental well-being, and social activities [58]. Often, a lack of self-knowledge, poor coping skills, and a greater sensitivity to being influenced by external events leads to a mismanagement of events and the feeling of losing one’s goals [59]. Moreover, some researchers have suggested a strong association between aggression and anxiety [60,61]; it could be hypothesized that having adaptive responses to anxiety to cope with the personal stress caused by restrictive measures can also be useful in dealing with the spread of the epidemic [62,63,64].

Important results were obtained for the two PANAS dimensions. Negative emotions can easily lead to a wide range of both individual and social problems, increasing the susceptibility to stress-related disorders [65,66,67]. Conversely, experiencing positive emotions promotes functional emotional control to cope with highly stressful situations [59]. Our findings related to both the PA and NA dimensions seemed to be consistent with the progression of the pandemic and the strengthening of the restrictive measures. This can be considered a physiological trend and adaptation to the stressful situation (i.e., cognitive reappraisal and acceptance) [68]. Furthermore, the emotional flatness reported in the middle phases seems to be in line with the psychological hibernation theory proposed by Sandal et al. [69]. A state of psychological hibernation may be useful in coping with the prolonged exposure to stressful stimuli and be used as an adaptive response; in particular, the ability to mentally “switch off” appears to be useful in achieving psychological detachment from a stressful situation.

Peak changes occurred at T3 for emerging adults, and the middle part of the lockdown was characterized by a sharp increase in the perceived stress levels (as shown for the PSS data). Our findings showed the cumulative exhaustion experienced by emerging adults throughout the period of interest; the prolonged confinement caused by restrictive COVID-19 measures imposed by governments might have acted as a chronic stressor, increasing individuals’ perceived distress after extended exposure. The general trends of the PSS scores throughout the study in all the different sessions seem to be explained by the model of the general adaptation syndrome [26]. Everything life-threatening may result in both stress and adaptive responses, making adaptability and resilience fundamental for facing stressful situations, in different life contexts [26,70].

The results suggest different effects: a general increase in the anxiety experienced by individuals during the lockdown period and in perceived stress leading to emotional “numbing” as the confinement progressed. Studies investigating stress-related conditions have already highlighted similar emotional effects in different but related situations, from traumatic events [71,72] to prolonged permanence in confined and extreme environments [69]. This interpretation is also in line with the findings describing a greater reliance on avoidant coping strategies during the lockdown.

The time-dependent effects on the perceived stress measures were further specified by their interactions with the age of participants; indeed, our results showed significantly lower mean scores for the two older groups than for emerging adults. This finding seems to be in line with the strength and vulnerability integration model (SAVI) [73], which suggests that older adults often develop strengths and greater resilience through a lifetime of experiences and are often better able to cope with challenges than younger adults. However, in some circumstances, if the challenges become too great, they may be at risk of adverse effects; in fact, when cognitive–behavioral strategies cannot be used, age-related vulnerabilities lead to greater physiological reactivity and poorer recovery, making it more difficult to cope with and mitigate stressful experiences [74,75]. This interpretation is in line with our results, which showed that the PSS scores of all the age groups were convergent at the FU. This result can also be attributed to the period at which the FU was carried out (i.e., September). In this period, an increasing number of new infections may have led to new concerns after a period of loosening of the restrictive measures during the summer that may have “buffered” the negative effect of daily stress by promoting more adaptive coping strategies [21].

Individuals with high levels of coping strategies perceive uncertain situations as less stressful and, thus, adapt quickly to new life conditions. Several studies have also suggested the central role of individual resilience and well-being as predictors of the better management of hazards, such as the COVID-19 crisis [76,77]. Being less familiar with dangerous situations could generate a higher stress level, considering the “danger” (i.e., COVID-19) as a difficult challenge to overcome. Additionally, basic resilience and perceived well-being act as protective factors that improve the ability to move forward and overcome the crisis.

In addition, while adults stayed in their homes, young people living alone (i.e., off-site students or emerging/young adults working away from home), for the most part, moved into their parents’ houses during the lockdown period, which can be considered a stressor [78]. Studies have shown that emerging adults reported higher rates of “staying at home” during the day, with a consequent decrease in their social interactions; although their generation makes much use of social media communication, a decrease in face-to-face interactions can be experienced as stressful [79,80]. Some studies have hypothesized that easy and more frequent access to the web, media, and news channels by emerging adults could lead to higher levels of anxiety, triggering a greater response to stress [11,50].

The results obtained from the “future” of the Time Perspective Inventory (STPI) Short Form and the Positivity Scale (P Scale) showed a comparable trend; the values regarding the “future” dimension of the STPI gradually decreased during the sessions, reaching a minimum peak at T4 and increasing significantly at the FU. A positive orientation toward the future is an important protective factor against stress [81]. A positive orientation has been negatively associated with a negative affect, being instead an important factor of general well-being and social adjustment [82]. Two main factors of positive orientation, optimism and hope, both with distinct characteristics, have shown effects on different chronic diseases [83]. Our results show that the factors such as social isolation, staying at home, and close cohabitation have important effects on the positive thinking of individuals.

During the lockdown, all nonessential businesses and industries were closed, and the mobility of people was restricted, resulting in a situation never experienced before and, thus, the generation of different adaptive responses in people [12,84]. However, the novelty of the situation and the uncertainty it produced at many different levels—for instance, on the perception of health and economic safety—might have accompanied individuals’ emotional adaptations with a modulation of their perceptions about the future. In this regard, future perspectives reflect crucial coping aspects such as resilience and the ability to think positively, configuring themselves as a powerful protective ability in emergency situations. In addition, some peculiar elements related to emerging adults can be considered; in particular, the lack of a regular daily routine during quarantine was most common in the younger group, which was mostly composed of students, and more conditioned by the disruption of their main activities, causing uncertainty for the future [85,86].

This study had some limitations that should be mentioned. First, the survey was conducted online, limiting the sample to only those who had access to the internet. However, online recruitment helped us to collect data from a different sample, given the restriction of physical mobility because of the lockdown. Second, the results may not have been directly applicable to the Italian population because of the limited representativeness; age-based sampling led to different distributions of individual characteristics compared to those of the Italian population. Another limitation was that the levels of the psychological impacts (e.g., anxiety, depression, and stress) were self-reported, and self-reported results may not always align with objective assessments by mental health practitioners. Finally, the sudden occurrence of the pandemic and the restrictive measures did not allow the collection of a pre-lockdown baseline; although the T1 data were collected in an early phase of the lockdown, the absence of “baseline” individual well-being information in the weeks before the lockdown could raise a concern of confounders in the trends in individual well-being over time that coincided with, but were not caused by, the COVID-19 lockdown. Despite these limitations, the strength of our study lied in its longitudinal nature, which highlighted the trend of psychological outcomes during the lockdown in Italy. We measured the outcomes at five time points: four time points during the lockdown phase and one time point six months after the end of the lockdown, allowing us to assess whether the psychological outcomes changed at different critical moments. Moreover, the experimental design acted as an obstacle in achieving a very large sample size. For instance, we registered a large number of dropouts. Despite the limited sample size, we found evidence highlighting the age-based disparities in the effects of the lockdown, probably because of different interpretations of the pandemic circumstances.

## 5. Conclusions

The present longitudinal study closely monitored quarantine responses during the Italian lockdown in terms of changes in people’s emotional state, perceived stress, and future perspective. Scores related to the emotional states (aggressiveness, anxiety, and positive and negative emotions) were generally higher than those compatible with an “alarm reaction” phase. Otherwise, the scores of perceived stress generally increased in the subsequent phases (resistance and exhaustion), showing a mild recovery several months later (post-lockdown), especially in emerging adults.

## Figures and Tables

**Table 1:** Descriptive statistics.

		*N*	%
Sex	Male	32	26.0%
	Female	91	74.0%
Education	Middle school	10	8.1%
	High school	52	42.3%
	Bachelor’s degree	36	29.3%
	Second degree	25	20.3%
Marital Status	Single	88	71.5%
	Married	30	24.4%
	Separated	3	2.4%
	Divorced	2	1.6%
Occupation	Student	59	48.0%
	Police Forces	1	0.8%
	Healthcare Profession	4	3.3%
	Employed	18	14.6%
	Unemployed	1	0.8%
	Worker	8	6.5%
	Freelancer	12	9.8%
	Homemaker	4	3.3%
	Teacher	5	4.1%
	Retiree	5	4.1%
	Other	6	4.9%
House Sqm	−40 sqm	3	2.4%
	from 40 to 80 sqm	40	32.5%
	+80 sqm	80	65.0%
Outdoor spaces	No	5	4.1%
	Balcony	51	41.5%
	Rooftop	15	12.2%
	Garden	33	26.8%
	Courtyard	19	15.4%
Live with other people	Yes	117	95.1%
	No	6	4.9%
Domestic animal	Yes	59	48.0%
	No	64	52.0%

**Table 2:** Descriptive statistics on the emotional state variables.

Variables	Age Group (*N*)	T1 M (SD)	T2 M (SD)	T3 M (SD)	T4 M (SD)	FU M (SD)
STAI-Y1	18–23 (46)	50.78 (10.92)	45.85 (10.49)	47.57 (12.36)	46.78 (10.04)	41.70 (13.01)
	24–36 (38)	43.26 (12.46)	41.00 (8.91)	43.97 (10.75)	43.32 (11.58)	39.58 (8.78)
	+36 (39)	41.59 (10.34)	41.10 (11.39)	41.59 (10.59)	42.21 (12.69)	38.72 (12.47)
	Total (123)	45.54 (11.89)	42.85 (10.51)	44.56 (11.52)	44.26 (11.49)	40.10 (11.66)
STAI-Y2	18–23 (46)	50.09 (10.61)	-	-	-	48.59 (10.73)
	24–36 (38)	44.13 (9.19)	-	-	-	42.71 (8.19)
	+36 (39)	39.95 (8.68)	-	-	-	40.49 (10.52)
	Total (123)	45.03 (10.43)	-	-	-	44.20 (10.47)
PANAS-PA	18–23 (46)	29.07 (8.53)	30.07 (8.17)	29.30 (8.63)	30.17 (6.67)	33.02 (8.26)
	24–36 (38)	30.76 (7.70)	31.87 (7.19)	30.79 (7.83)	30.21 (7.93)	35.24 (6.63)
	+36 (39)	33.97 (6.20)	32.95 (5.77)	31.79 (6.76)	30.46 (7.98)	34.41 (8.68)
	Total (123)	31.15 (7.81)	31.85 (7.25)	30.55 (7.84)	30.28 (8.16)	34.15 (7.93)
PANAS-NA	18–23 (46)	26.22 (7.36)	24.83 (7.53)	25.30 (7.45)	23.93 (7.32)	22.83 (8.16)
	24–36 (38)	24.82 (8.10)	22.45 (7.77)	23.97 (7.74)	23.76 (8.87)	20.87 (7.16)
	+36 (39)	24.00 (7.05)	21.46 (7.58)	22.41 (8.26)	22.64 (9.05)	20.10 (7.74)
	Total (123)	25.08 (7.50)	23.02 (7.70)	23.98 (7.83)	23.47 (8.33)	21.36 (7.76)
AQ-PA	18–23 (46)	25.52 (4.05)	24.54 (4.85)	24.24 (4.29)	23.65 (4.37)	24.30 (4.52)
	24–36 (38)	23.11 (4.76)	22.13 (4.72)	22.61 (3.51)	22.26 (3.70)	21.16 (4.60
	+36 (39)	21.03 (4.89)	20.10 (5.32)	21.00 (4.99)	19.64 (5.21)	20.38 (5.54)
	Total (123)	23.35 (4.89)	22.39 (5.26)	22.71 (4.48)	21.95 (4.74)	22.09 (5.15)
AQ-VA	18–23 (46)	17.46 (4.88)	17.22 (5.51)	16.89 (5.41)	16.65 (5.31)	17.35 (5.23)
	24–36 (38)	16.61 (4.54)	16.08 (4.62)	16.13 (4.72)	16.84 (4.58)	16.11 (4.99)
	+36 (39)	15.64 (5.25)	14.85 (5.41)	13.85 (4.44)	13.87 (4.50)	14.85 (4.56)
	Total (123)	16.62 (4.92)	16.11 (5.27)	15.69 (5.04)	15.89 (4.99)	16.17 (5.02)
AQ-HO	18–23 (46)	21.07 (5.05)	21.35 (6.21)	21.52 (6.17)	21.72 (5.46)	20.33 (4.87)
	24–36 (38)	19.39 (5.33)	19.13 (5.85)	19.63 (6.07)	19.50 (5.63)	19.42 (5.33)
	+36 (39)	17.67 (5.88)	17.21 (6.19)	17.03 (5.02)	16.85 (5.82)	17.13 (5.44)
	Total (123)	19.47 (5.55)	19.35 (6.28)	19.51 (6.05)	19.49 (5.94)	19.03 (5.33)

Note: STAI-Y1 = State Anxiety Inventory, STAI-Y2 = Trait Anxiety Inventory, PANAS-PA = PANAS Positive Affect, PANAS-NA = PANAS Negative Affect, AQ-PA = Physical Aggressivity of Aggression Questionnaire, AQ-VA = Verbal Aggressivity of Aggression Questionnaire, and AQ-HO = Hostility of Aggression Questionnaire.

**Table 3:** Descriptive statistics on the perceived stress variables.

Variables	Age Group (*N*)	T1 M (SD)	T2 M (SD)	T3 M (SD)	T4 M (SD)	FU M (SD)
SUDS	18–23 (46)	6.33 (1.96)	6.37 (2.03)	6.91 (1.86)	6.84 (2.12)	5.44 (2.25)
	24–36 (38)	5.71 (2.21)	5.87 (2.21)	6.00 (2.27)	5.90 (2.64)	5.00 (2.27)
	+36 (39)	5.09 (2.58)	5.60 (2.43)	5.27 (2.69)	5.80 (2.72)	5.38 (2.28)
	Total (123)	5.75 (2.29)	5.97 (2.22)	6.11 (2.36)	6.22 (2.51)	5.28 (2.26)
PSS-TOT	18–23 (46)	19.93 (4.88)	20.07 (6.09)	20.72 (6.29)	19.83 (5.09)	19.41 (2.62)
	24–36 (38)	16.71 (5.09)	16.42 (6.09)	17.82 (6.60)	17.32 (5.63)	19.05 (2.45)
	+36 (39)	13.77 (4.66)	14.59 (7.44)	13.10 (5.57)	13.56 (5.31)	19.31 (2.41)
	Total (123)	16.98 (5.48)	17.20 (6.90)	17.41 (6.90)	17.07 (5.90)	19.27 (2.49)
COPE-AV	18–23 (46)	23.11 (3.85)	-	-	-	22.67 (4.72)
	24–36 (38)	21.58 (4.57)	-	-	-	20.47 (3.85)
	+36 (39)	20.41 (3.40)	-	-	-	19.67 (3.73)
	Total (123)	21.78 (4.08)	-	-	-	21.04 (4.33)
COPE-AP	18–23 (46)	31.59 (5.39)	-	-	-	31.70 (5.16)
	24–36 (38)	31.24 (4.35)	-	-	-	31.24 (4.16)
	+36 (39)	32.05 (4.70)	-	-	-	31.21 (4.62)
	Total (123)	31.63 (4.84)	-	-	-	31.40 (4.67)
COPE-HU	18–23 (46)	4.52 (1.60)	-	-	-	4.17 (1.53)
	24–36 (38)	4.66 (1.67)	-	-	-	4.32 (1.56)
	+36 (39)	4.03 (1.60)	-	-	-	3.92 (1.51)
	Total (123)	4.41 (1.63)	-	-	-	4.14 (1.53)
COPE-RE	18–23 (46)	2.41 (0.78)	-	-	-	2.63 (1.06)
	24–36 (38)	2.76 (1.48)	-	-	-	2.66 (1.17)
	+36 (39)	3.36 (1.77)	-	-	-	3.18 (1.82)
	Total (123)	2.82 (1.42)	-	-	-	2.81 (1.39)

Note: PSS-TOT = Perceived Stress Scale Total score, SUDS = Subjective Units of Disturbance Scale, COPE-AV = Brief Cope Inventory Avoidance, COPE-AP = Brief Cope Inventory Approach, COPE-HU = Brief Cope Inventory Humor, and COPE-RE = Brief Cope Inventory Religion.

**Table 4:** Descriptive statistics on the future perspective variables.

Variables	Age Group (*N*)	T1 M (SD)	T2 M (SD)	T3 M (SD)	T4 M (SD)	FU M (SD)
P-SCALE	18–23 (46)	27.00 (5.80)	27.00 (5.52)	26.35 (5.36)	26.20 (5.34)	25.72 (6.30)
	24–36 (38)	28.92 (4.75)	28.55 (5.16)	28.05 (5.30)	27.39 (5.26)	28.66 (4.66)
	+36 (39)	29.08 (5.55)	28.79 (5.96)	28.05 (5.75)	27.49 (6.83)	28.74 (6.51)
	Total (123)	28.25 (5.46)	28.05 (5.57)	27.41 (5.49)	26.98 (5.82)	27.59 (6.04)
STPI-FUT	18–23 (46)	32.46 (6.81)	32.09 (6.37)	32.24 (6.37)	31.63 (6.79)	32.89 (5.89)
	24–36 (38)	31.97 (4.80)	32.32 (4.74)	32.63 (4.61)	31.87 (5.07)	33.61 (4.76)
	+36 (39)	34.36 (4.83)	33.79 (7.56)	34.00 (5.99)	33.54 (5.55)	34.18 (6.23)
	Total (123)	32.91 (5.69)	32.70 (6.34)	32.92 (5.76)	32.31 (5.93)	33.52 (5.66)
STPI-HED	18–23 (46)	23.22 (4.03)	24.24 (3.43)	23.35 (3.62)	23.28 (4.11)	23.67 (3.29)
	24–36 (38)	23.87 (3.52)	23.82 (3.83)	23.53 (3.92)	23.47 (3.76)	22.87 (3.85)
	+36 (39)	22.59 (3.42)	22.56 (3.89)	22.08 (3.22)	23.31 (3.28)	22.87 (3.22)
	Total (123)	23.22 (3.69)	23.58 (3.74)	23.00 (3.62)	23.35 (3.72)	23.17 (3.45)
STPI-FAT	18–23 (46)	14.26 (3.85)	13.89 (3.39)	14.22 (3.49)	14.07 (3.39)	13.91 (3.49)
	24–36 (38)	13.97 (4.04)	14.45 (4.41)	14.37 (3.70)	14.26 (3.16)	14.13 (3.60)
	+36 (39)	15.16 (3.66)	14.97 (3.65)	15.16 (3.37)	15.03 (3.76)	15.05 (3.31)
	Total (123)	14.45 (3.85)	14.40 (3.48)	14.56 (3.51)	14.43 (3.44)	14.34 (3.48)

Note: P_SCALE = Positivity Scale, STPI-FUT = Future dimension of Stanford Time Perspective Inventory, STPI-HED = Hedonistic dimension of the Stanford Time Perspective Inventory, and STPI-FAT = Fatalistic dimension of the Stanford Time Perspective Inventory.

## Data Availability

This study was not preregistered, and the data that support the findings of this study are available on request from the corresponding author (A.Q.). The data are not publicly available due to restrictions, e.g., containing information that could compromise the privacy of research participants.
